# RETRACTION: The Cotransplantation of Olfactory Ensheathing Cells With Bone Marrow Mesenchymal Stem Cells Exerts Antiapoptotic Effects in Adult Rats After Spinal Cord Injury

**DOI:** 10.1155/sci/9805310

**Published:** 2026-07-08

**Authors:** Stem Cells International

RETRACTION: S. Wu, G. Cui, H. Shao, Z. Du, J. C. Ng, and C. Peng, “The Cotransplantation of Olfactory Ensheathing Cells With Bone Marrow Mesenchymal Stem Cells Exerts Antiapoptotic Effects in Adult Rats After Spinal Cord Injury,” *Stem Cells International* 2015, no. 1 (2015): 1–13, https://doi.org/10.1155/2015/516215.

The above article, published online on 29 July 2015 in Wiley Online Library (wileyonlinelibrary.com), has been retracted by agreement between the Journal Chief Editor, Renke Li; and John Wiley & Sons Ltd.

The retraction has been agreed following an investigation into concerns initially raised on PubPeer [1]. Abnormal similarities were identified between several cell staining images that were meant to represent different experimental conditions:

• In Figure 4, D2 appears abnormally similar to the right side of E1 rotated 90 degrees anti‐clockwise
• In Figure 5, D2 appears abnormally similar to the left side of C2 rotated 90 degrees anti‐clockwise

The publisher verified the concerns and found additional areas of overlap:

• In Figure 4, the bottom‐left section of B1 appears abnormally similar to the left side of C2 rotated 90 degrees anti‐clockwise
• In Figure 5, there appears to be a small area of overlap between the bottom of A1 and the top of A2

The authors were contacted but did not provide a sufficient explanation, and they were unable to provide their original raw data and figures when they were asked to address these concerns. As a result, the data and conclusions of this article are considered unreliable. Therefore, the article is retracted.

The authors did not respond when they were notified of the decision to retract.
